# Cannabinoid and Cannabinoid-Related Receptors in the Myenteric Plexus of the Porcine Ileum

**DOI:** 10.3390/ani11020263

**Published:** 2021-01-21

**Authors:** Andrea Toschi, Giorgia Galiazzo, Andrea Piva, Claudio Tagliavia, Gemma Mazzuoli-Weber, Roberto Chiocchetti, Ester Grilli

**Affiliations:** 1 Department of Veterinary Medical Sciences (UNI EN ISO 9001:2008), University of Bologna, Via Tolara di Sopra, 50, 40064 Ozzano dell’Emilia, Italy; andrea.toschi5@unibo.it (A.T.); giorgia.galiazzo2@unibo.it (G.G.); andrea.piva@unibo.it (A.P.); claudio.tagliavia2@unibo.it (C.T.); ester.grilli@unibo.it (E.G.); 2 R&D Division, Vetagro S.p.A., via Porro 2, 42124 Reggio Emilia, Italy; 3 Institute for Physiology and Cell Biology, University of Veterinary Medicine Hannover, Foundation, Bischofsholer Damm 15, 30173 Hannover, Germany; Gemma.Mazzuoli-Weber@tiho-hannover.de; 4 R&D Division, Vetagro, Inc., 116 W. Jackson Blvd., Suite #320, Chicago, IL 60604, USA

**Keywords:** CB1R, CB2R, TRPV1, TRPA1, 5-HT1aR, endocannabinoid system, immunohistochemistry, pig, enteric nervous system

## Abstract

**Simple Summary:**

The endocannabinoid system (ECS) has opened the door to novel therapeutical approaches targeting cancer, pain, anxiety, stress, and inflammatory diseases. The ECS is ubiquitously expressed in almost all members of *Animalia*, but its precise localization outside the central nervous system is still under investigation. In this study, the localization of the main and related cannabinoid receptors in the myenteric plexus of the porcine ileum was immunohistochemically analyzed. The myenteric plexus neurons were found to be positive for cannabinoid receptor 1 (CB1R) and the cannabinoid-related receptors transient potential vanilloid receptor 1 (TRPV1), transient potential ankyrin receptor 1 (TRPA1), and serotonin receptor 5-HT1a (5-HT1aR). In addition, the ECS receptors were also located on nerve fibers, the *tunica muscularis*, and the endothelium. The wide distribution of cannabinoid and cannabinoid-related receptors in the myenteric plexus provides the anatomical basis for additional investigation, suggesting the possible role of the ECS in treating pathological conditions in livestock.

**Abstract:**

An important piece of evidence has shown that molecules acting on cannabinoid receptors influence gastrointestinal motility and induce beneficial effects on gastrointestinal inflammation and visceral pain. The aim of this investigation was to immunohistochemically localize the distribution of canonical cannabinoid receptor type 1 (CB1R) and type 2 (CB2R) and the cannabinoid-related receptors transient potential vanilloid receptor 1 (TRPV1), transient potential ankyrin receptor 1 (TRPA1), and serotonin receptor 5-HT1a (5-HT1aR) in the myenteric plexus (MP) of pig ileum. CB1R, TRPV1, TRPA1, and 5-HT1aR were expressed, with different intensities in the cytoplasm of MP neurons. For each receptor, the proportions of the immunoreactive neurons were evaluated using the anti-HuC/HuD antibody. These receptors were also localized on nerve fibers (CB1R, TRPA1), smooth muscle cells of *tunica muscularis* (CB1R, 5-HT1aR), and endothelial cells of blood vessels (TRPV1, TRPA1, 5-HT1aR). The nerve varicosities were also found to be immunoreactive for both TRPV1 and 5-HT1aR. No immunoreactivity was documented for CB2R. Cannabinoid and cannabinoid-related receptors herein investigated showed a wide distribution in the enteric neurons and nerve fibers of the pig MP. These results could provide an anatomical basis for additional research, supporting the therapeutic use of cannabinoid receptor agonists in relieving motility disorders in porcine enteropathies.

## 1. Introduction

The network of sensory neurons, motor neurons, interneurons, and glial cells embedded in the gut walls is called the enteric nervous system (ENS) [1]. The ENS is responsible for controlling various functions of the gastrointestinal tract (GIT) such as motility, absorption, and secretion in physiological and pathological conditions [2]. There is a strict interaction between the ENS and the central nervous system (CNS), with a bidirectional information flow between these two systems; however, the ENS can control digestive functions independently of the CNS [3]. The ENS also cooperates with the immune and endocrine systems by adapting nutrient absorption depending on the condition of the gut, thereby preserving mucosal barrier functionality [3].

The endocannabinoid system (ECS) is constituted of three fundamental components: receptors, signaling molecules, and the enzymes responsible for ligand biosynthesis and degradation. It typically comprises the prototypical cannabinoid receptors types 1 and 2 (CB1R and CB2R), endocannabinoids anandamide (AEA) and 2-arachidonylglycerol (2-AG), and the enzymes involved in their biosynthesis and degradation [4,5,6,7]. The ECS is typically localized at the CNS level [8]. CB1R was proven to be the most widely expressed receptor protein from the G protein-coupled receptors (GPCRs) family in the brain, mainly in the basal ganglia, hippocampus, olfactory bulb, and cerebellum [9]. In contrast, CB2R is mainly expressed in immune tissues such as the microglia, leukocytes, and cells of macrophage lineage [10,11]. The broad localization of the CB1R in the CNS represents a limit to its potential as a pharmacological target for CNS pathologies due to the undesired psychotropic side effects related to its activation from agonists and antagonists [12]. On the other hand, CB2R may constitute a promising pharmacological target for inflammatory disorders, thanks to its anti-inflammatory properties [13]. It has recently been clarified that the localization of the ECS is not limited only to the CNS since it was found ubiquitously expressed throughout the body, serving a multiplicity of physiological roles including the regulation of gastrointestinal functions [14,15]. In particular, the ECS is supposed to regulate gastrointestinal secretion and motility via the ENS [16]. Various studies have suggested a possible implication of CB1R and CB2R in inflammatory bowel disease (IBD), exerting a protective effect, thus suggesting the potential of pharmacological agents capable of targeting and modulating these pathways [15,17,18]. Moreover, additional cannabinoid-related receptors and endocannabinoid-like molecules may also be involved. In particular, among the secondary receptors belonging to the ECS are found G-protein coupled receptors (GPRs), transient receptor potential (TRP) channels, serotonin (5-HT) receptors, and nuclear peroxisome proliferator-activated receptors (PPARs) [19]. In particular, TRP channels are sensitive to harmful stimuli, pungent compounds, acid, temperature, and inflammation mediators, qualifying these receptors as being suitable candidates and novel targets for gastrointestinal pain [20].

Concerning the endocannabinoid-like mediators, growing interest is driven by palmitoylethanolamide (PEA) and cannabidiol (CBD) [6,21]. Growing research regarding this topic indicates that activation of the cannabinoid and cannabinoid-related receptors, mediated by endogenous or plant-derived cannabinoids, may influence GIT motility and secretion, with a reduction in inflammation and visceral pain [17,22,23,24,25,26,27].

To the authors’ knowledge, only a few studies have described the presence of the ECS in the porcine GIT, limited to the mucosa [28] or, in the ENS, only to CB1R [29]. For this reason, the aim of this study was to immunohistochemically characterize the distribution of the canonical cannabinoid receptors CB1R and CB2R, and the cannabinoid-related receptors TRP vanilloid 1 (TRPV1), TRP ankyrin 1 (TRPA1), and 5-HT1a serotonin receptor (5-HT1aR) in the myenteric plexus (MP) of the pig ileum.

## 2. Materials and Methods 

### 2.1. Animals 

Intestinal tissues were collected from six pigs at the slaughterhouse. All animals were 7-month-old genetic hybrids (Landrace × Large White). The animals did not have a history of gastrointestinal disorders and did not show gross alterations of the gastrointestinal wall. 

Italian legislation (D. Lgs. n. 26/2014), according to Directive 2010/63/EU of the European Parliament and the Council of 22/09/2010 regarding the protection of animals used for scientific purposes, does not require any approval by the appropriate authorities or ethics committees since this research did not influence any therapeutic decisions.

### 2.2. Tissue Collection

The ileum was harvested within 30 min from the animals’ deaths and was longitudinally opened along the mesenteric border. The tissues were then washed in phosphate-buffered saline (PBS), fixed, and processed to obtain longitudinal (2.0 cm × 0.5 cm) and tangential cryosections (2.0 cm × 1.0 cm), which were later processed for immunohistochemistry, as previously described [30].

### 2.3. Immunofluorescence

After hydration in PBS, the cryosections were processed for immunostaining. To prevent non-specific bindings, the cryosections were incubated in a solution containing 20% normal donkey serum (Colorado Serum Co., Denver, CO, USA), 0.5% Triton X-100 (Sigma Aldrich, Milan, Italy, Europe), and bovine serum albumin (1%) in PBS for 1 h at room temperature (RT). The cryosections were then incubated in a humid chamber overnight at RT, and single or double immunostaining was carried out. In the single immunostaining, the cryosections were incubated with only one of the primary antibodies (Table 1) directed against the cannabinoid and cannabinoid-related receptors. For double immunostaining, the cryosections were incubated with a cocktail of primary antibodies (Table 1). Since double immunostaining was carried out to identify the enteric neurons, the cryosections were co-incubated with one of the anti-cannabinoid receptors or anti-cannabinoid-related antibodies and the anti-HuC/HuD antibody. All the primary antibodies were diluted in 1.8% NaCl in 0.01 M PBS containing 0.1% sodium azide. After washing the cryosections in PBS (3 × 10 min), they were incubated for 1 h at RT in a humid chamber with the secondary antisera (Table 2) diluted in PBS. The cryosections were then washed in PBS (3 × 10 min) and mounted in buffered glycerol at pH 8.6 with 4′,6-diamidino-2-phenylindole (DAPI) (Santa Cruz Biotechnology, Santa Cruz, CA, USA).

The proportion of neurons that were HuC/HuD immunoreactive and that were also immunoreactive for CB1R, CB2R, TRPV1, TRPA1, and 5-HT1aR was determined by examining fluorescently labelled, double-stained preparations. The neurons were first identified by the presence of a fluorophore labeling one antigen (HuC/HuD), and the microscope filter was subsequently switched to determine whether or not the neuron expressed a second antigen (CB1R, CB2R, TRPV1, TRPA1, and 5-HT1aR), identified with a different-colored fluorophore. In doing so, the proportion of neurons labeled for pairs of antigens was determined.

A minimum of one hundred HuC/HuD immunoreactive MP neurons was counted for each marker expressed by nerve cell bodies. Data were collected from preparations obtained from at least three animals (*n* = 5). The percentages of immunoreactive neurons were expressed as mean ± standard deviation.

### 2.4. Specificity of the Primary Antibodies

CB1R, the synthetic peptide MSVSTDTSAEAL, corresponding to carboxy-terminal amino acids 461-472 of human CB1R, was used as an immunogen to obtain the anti-CB1R antiserum. The homology between the full amino acid sequences of the pig (F1S0E6) and the human (P21554) CB1R was 97.9% (https://blast.ncbi.nlm.nih.gov/Blast.cgi); correspondence with the specific sequence of the immunogen was 100%. Therefore, the antibody anti-CB1R should also recognize the same receptor in pig. Since this antibody is human specific, it was applied on a submucosal wholemount preparation of human descending colon as a positive control, having previously obtained donor consent. The wholemount preparation was prepared and analyzed using pre-validated immunohistochemical protocols [31].

CB2R, the synthetic peptide conjugated to keyhole limpet hemocyanin (KLH) derived from within residues 200–300 of rat CB2, was used as an immunogen to obtain the rabbit ant-CB2R antibody (ab45942). The homology between the full amino acid sequences of pig (I3LUS5) and rat CB2R (Q9QZN9) was 76.3%; the correspondence with the specific sequence of the immunogen was 76%. The amino acid sequence 302–360 of CB2 of human origin (P34972) was used as an immunogen to obtain the mouse anti-CB2R antibody (sc-293188). The homology between the full amino acid sequences of pig and human CB2R was 81.9%. 

TRPV1, the peptide (C)EDAEVFK DSMVPGEK, corresponding to residues 824–838 of rat TRPV1, was used as an immunogen to obtain the anti-TRPV1 antibody. The homology between the full amino acid sequences of pig (A0A4X1UCR0) and rat (O35433) TRPV1 was 84.52% (https://blast.ncbi.nlm.nih.gov/Blast.cgi), and the correspondence with the specific sequence of the immunogen was 93%. However, this antibody was tested on the porcine nervous system (dorsal root ganglia) using western blot (Wb) analysis [32].

TRPA1, the synthetic peptide CEKQHELIKLIIQKME, corresponding to amino acids 1060-1075 of rat TRPA1, was used as an immunogen to obtain the anti-TRPA1 antibody. The alignment of the immunogen sequence with the target protein in the pig was 93% (https://blast.ncbi.nlm.nih.gov/Blast.cgi). It is plausible that the antibody anti-rat TRPA1 should also recognize the same receptor in the pig.

5-HT1aR, the synthetic peptide, corresponding to amino acids 100–200 (conjugated to keyhole limpet hemocyanin) of rat 5-HT1aR, was used as an immunogen to obtain the anti-5-HT1aR antibody. The alignment of the immunogen with the target protein sequence in the pig was 100% (https://blast.ncbi.nlm.nih.gov/Blast.cgi). Therefore, the antibody anti-rat 5-HT1aR should also recognize the same receptor in pig.

The suppliers of the anti-CB2R, -TRPV1, -TRPA1, and -5-HT1aR antibodies employed in the present study stated that they were rat specific; thus, for comparison purposes, the anti-CB2R and -TRPV1 antibodies were applied on the positive control tissues, in particular on wholemount preparations of rat ileum (authorization no. 112/2018-PR of 12 February 2018). Data related to the anti-TRPA1 and 5-HT1aR antibodies have recently been published [33].

### 2.5. Specificity of the Secondary Antibodies

The specificity of the secondary antibodies (Table 2) was tested by the absence of signal after the exclusion of the primary antibody on pig intestinal tissues.

### 2.6. Fluorescence Microscopy

Cryosections and wholemount preparations were examined by the same observer (Dr. R. Chiocchetti) using a Nikon Eclipse Ni microscope (Nikon Instruments Europe BV, Amsterdam, The Netherlands) equipped with the appropriate filter cubes to differentiate the fluorochromes used. The images were recorded using a Nikon DS-Qi1Nc digital camera and NIS Elements software BR 4.20.01 (Nikon Instruments Europe BV, Amsterdam, Netherlands). Enteric neuron counts were carried out at 40× magnification. Slight adjustments to contrast and brightness were made using Corel Photo Paint whereas the figure panels were prepared using Corel Draw (Corel Photo Paint and Corel Draw, Ottawa, ON, Canada).

## 3. Results

### 3.1. CB1R Immunoreactivity

**Figure 1:**
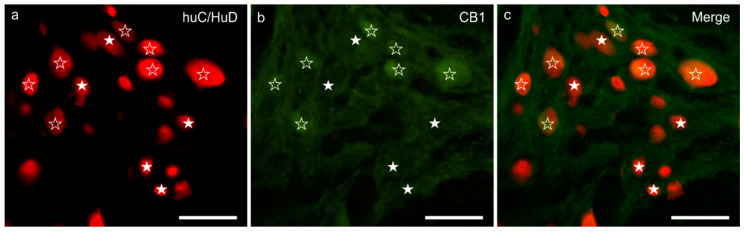
CB1 receptor immunoreactivity in the myenteric plexus of the pig ileum: (**a**) HuC/HuD immunoreactive neurons, (**b**) CB1 receptor immunoreactivity, (**c**) merge image. The open stars indicate HuC/HuD immunoreactive neurons co-expressing weak-to-moderate CB1 receptor immunoreactivity. The white stars indicate HuC/HuD immunoreactive neurons, which were CB1 negative. Scale bar: 50 µm.

Weak-to-moderate granular and diffuse CB1R immunoreactivity (CB1R-IR) was expressed by the cytoplasm of the MP neurons; the brightest CB1R immunoreactive neurons showed large dimensions and a smooth outline (Figure 1a–c). The percentages of HuC/HuD immunoreactive neurons co-expressing CB1R-IR was 57 ± 19% (377/713 cells counted, *n* = 5). Nerve fibers within the MP ganglia, distributed in the interganglionic strands and scattered within the muscular layers, showed weak CB1R-IR. Weak CB1R-IR was also observed in the smooth muscle cells of the *tunica muscularis* (longitudinal muscle layer, LML > circular muscle layer, CML) (data not shown).

Moderate CB1R-IR was expressed by the cytoplasm of the submucosal plexus neurons of the human colon (Figure S1).

### 3.2. CB2R Immunoreactivity

No immunolabeling was observed in the porcine MP with either of the anti-CB2 receptor antibodies. In the rat ileum, MP neurons expressed weak-to-moderate CB2R-IR (Figure S2).

### 3.3. TRPV1 Immunoreactivity

**Figure 2:**
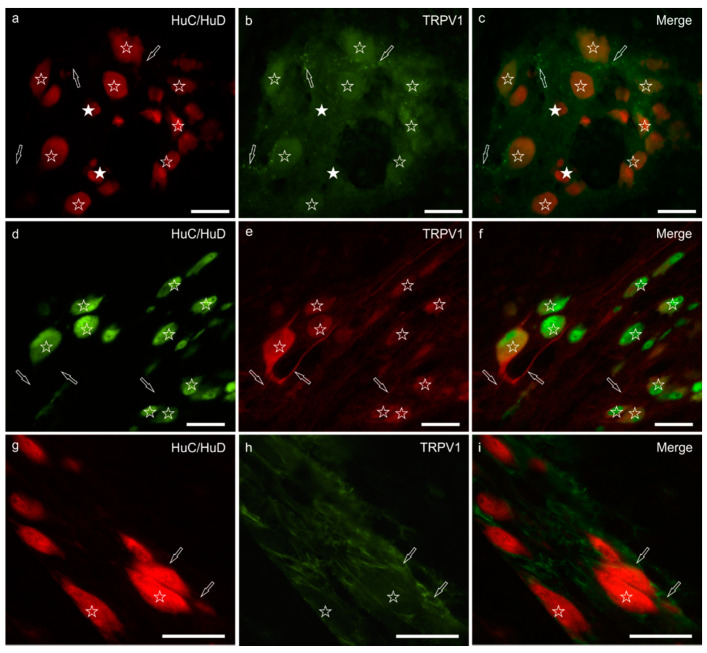
TRPV1 immunoreactivity in the myenteric plexus of the pig ileum: (**a**) HuC/HuD immunoreactive neurons, (**b**) TRPV1 immunoreactivity, (**c**) merge image. The open stars indicate the HuC/HuD immunoreactive neurons co-expressing moderate TRPV1 immunoreactivity. The white stars indicate HuC/HuD immunoreactive neurons, which were TRPV1 negative. The arrows indicate the TRPV1 immunoreactive varicosities encircling the neuronal cell bodies. (**d**) HuC/HuD immunoreactive neurons, (**e**) TRPV1 immunoreactivity, (**f**) merge image. The stars indicate the HuC/HuD immunoreactive neurons co-expressing moderate-to-bright TRPV1 immunoreactivity. The arrows indicate TRPV1 immunoreactive neuronal processes. (**g**) HuC/HuD immunoreactive neurons, (**h**) TRPV1 immunoreactivity, (**i**) merge image. The stars indicate two HuC/HuD immunoreactive myenteric plexus neurons co-expressing weak and diffuse TRPV1 immunoreactivity; the arrows indicate two perineuronal enteric glial cells expressing bright TRPV1 immunoreactivity. Scale bar: 50 µm.

Moderate-to-bright granular TRPV1-IR was expressed by the cytoplasm of the majority of the MP neurons (71 ± 14%; 462/602 cells counted, *n* = 5). The TRPV1 immunolabelling, which was mainly confined to the cell bodies of neurons showing a smooth outline, was more intense in neurons of large dimensions (Figure 2a–c) whereas it was very faint or undetectable in neurons of small dimensions. However, TRPV1 immunoreactive nerve processes arising from large neurons were also visible (Figure 2d–f). Few TRPV1 immunoreactive nerve fibers were seen in the MP ganglia, in the interganglionic nerve strands and within the muscular layers; nevertheless, bright and small TRPV1 immunoreactive varicosities were seen in the neuropil of the ganglia and around some MP neurons. In one subject, TRPV1 was also brightly expressed by the enteric glial cells (Figure 2g–i). Moderate TRPV1-IR was expressed by the endothelial cells of thin blood vessels (capillaries) distributed in the *tunica muscularis* (data not shown). In the rat ileum, MP and enteric glial cells (EGCs) expressed TRPV1-IR (EGCs > neurons) (Figure S3).

### 3.4. TRPA1 Immunoreactivity

**Figure 3:**
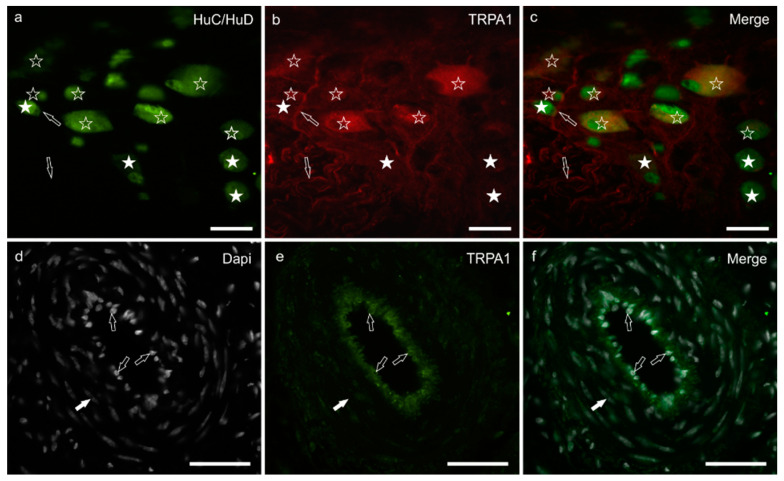
TRPA1 immunoreactivity in the myenteric plexus and blood vessels of the *tunica muscularis* of the pig ileum: (**a**) HuC/HuD immunoreactive neurons, (**b**) TRPA1 immunoreactivity, (**c**) merge image. The open stars indicate HuC/HuD immunoreactive neurons co-expressing moderate-to-bright TRPA1 immunoreactivity. The white stars indicate HuC/HuD immunoreactive neurons, which were TRPA1 negative. The arrows indicate a TRPA1 immunoreactive neuronal process. (**d**) Dapi stained nuclei of endothelial cells and vascular smooth muscle cells, (**e**) TRPA1 immunoreactivity, (**f**) merge image. The open arrows indicate the Dapi stained nuclei of endothelial cells expressing bright TRPA1 immunoreactivity. The white arrow indicates the elongated Dapi stained nucleus of one smooth muscle cell of the arterial *tunica media*. Scale bar: 50 µm.

Diffuse and moderate cytoplasmic TRPA1-IR was shown by a large percentage of MP neurons (66 ± 23%; 336/527 cells counted, *n* = 5) and was brighter in the cytoplasm of large neurons (Figure 3a–c). Nerve fibers within the ganglia and those distributed along the nerve strands and musculature showed moderate TRPA1-IR (data not shown). Bright TRPA1-IR was displayed by the endothelial cells of the blood vessels (Figure 3d–f). 

Transient potential ankyrin receptor 1 immunoreactivity was also expressed by the MP neurons in the ileum of a control rat [33].

3.5. 5-HT1aR Immunoreactivity

**Figure 4:**
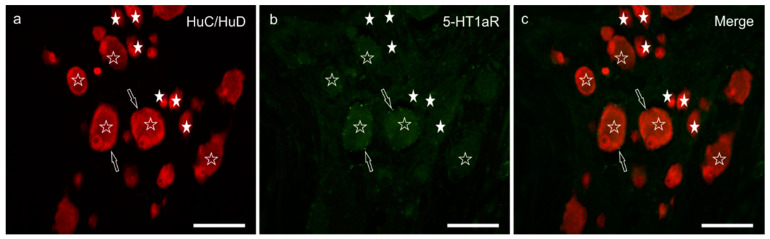
5-HT1a receptor immunoreactivity in the myenteric plexus of the pig ileum: (**a**) HuC/HuD immunoreactive neurons, (**b**) 5-HT1a receptor immunoreactivity, (**c**) merge image. The open stars indicate HuC/HuD immunoreactive neurons co-expressing weak 5-HT1a receptor immunoreactivity. The white stars indicate the HuC/HuD immunoreactive neurons, which were 5-HT1a receptor negative. The arrows indicate 5-HT1a receptor immunoreactive varicosities encircling the neuronal cell bodies. Scale bar: 50 µm.

Weak and diffuse 5-HT1aR immunolabelling was expressed by approximately half the MP neurons (51 ± 6%; 345/682 cells counted, *n* = 5) (Figure 4a–c). In general, neurons of large dimensions showed brighter immunofluorescence. The 5-HT1aR-IR was expressed by nerve varicosities. Weak 5-HT1aR-IR was displayed by the smooth muscle cells of the blood vessels and the *tunica muscularis* (data not shown).

The 5-HT1aR was also expressed by MP neurons in the rat ileum [33]. 

The results of the cellular distribution and intensity of immunolabeling in the pig ileum are summarized in semiquantitative Table 3.

## 4. Discussion

In the GIT, cannabinoid receptors regulate motility, secretion, emesis, food intake, and inflammation [15,17,24,26]. In this paper, the authors focused their attention on the presence of the ECS in the ileal MP of pigs, with particular emphasis on both the cannabinoid receptors, namely CB1R and CB2R, and the cannabinoid-related receptors TRPV1, TRPA1, and 5-HT1aR by carrying out immunohistochemical analysis.

The observation of the CB1R in the MP neurons and nerve fibers in the porcine ileum is consistent with the findings of Kulkarni-Narla and Brown [29]. The expression of CB1R-IR in the enteric neurons has been observed in many other species such as rodents, ferrets, dogs, cats, and humans [33,34,35,36,37]. Previous studies regarding various species including pigs have indicated that CB1R immunoreactive neurons show a cholinergic phenotype, exerting an inhibitory effect on MP cholinergic neurotransmission [29,38,39,40,41].

The evaluation of the proportion of myenteric CB1R immunoreactive neurons of the pig is not comparable with other data available in the literature. However, the percentage of CB1R immunoreactive neurons (57 ± 19%) observed in the present study was similar to the percentage of cholinergic neurons observed in the guinea-pig (approximately 80%; [42]), but was greater than the proportions of the ChAT-IR neurons counted in other species such as horses (64%; [43]), sheep (62%; [44]) or pigs (58%; [45]). In effect, Brehmer and colleagues [45] observed that there was a subclass of enteric cholinergic neurons, which could be identified only by the use of the antibody directed against the peripheral form of ChAT (i.e., pChAT). Thus, the percentage of cholinergic neurons of the pig ileum should be greater than that identified only with the anti-ChAT antibody. However, the great percentage of CB1R immunoreactive neurons observed in the present study indicated that this receptor might also be expressed by other neuronal subpopulations as well as by the cholinergic neurons. The influence on intestinal motility and contractility mediated by cannabinoids might be confirmed by the presence of CB1R on the smooth muscle cells, suggesting a direct muscular mechanism of cannabinoids [41]. 

CB2R is mainly expressed in immune tissues and cells of macrophage lineage [11]. The lack of immunoreactivity to the CB2R in enteric neurons is in line with the results obtained in the MP of dogs and cats [33,37] in contrast with rats (present study) and mice [25]. In the pig ileum, the lack of results could also depend on the low homology between the full amino acid sequences of pig and rat CB2R (76%), and pig and human CB2R (81.9%), original immunogens of the antibodies employed in the present study.

Transient potential vanilloid receptor 1 is a non-selective cation channel expressed by peptidergic and non-peptidergic nociceptors in rodents and large mammals [32,46,47]; TRPV1-IR was observed in the MP neurons of the pig (and rat) ileum, according to Poonyachoti et al. [48], who indicated that the majority of TRPV1 immunoreactive neurons were cholinergic. The expression of TRPV1-IR in the intramural neurons is a matter of debate. In fact, there are numerous investigations reporting TRPV1-IR expressed by extrinsic sensory neurons [49,50,51,52,53,54,55,56]. However, there are publications supporting the expression of TRPV1 immunolabelling in intramural neurons of different mammalian species [29,35,48,53,56,57,58,59] as has been shown in the present study. In addition, functional studies have supported the existence of enteric TRPV1 immunoreactive neurons in mice [60] and pigs [61].

The use of different anti-TRPV1 antibodies seems to be the reason for the discrepancy in the expression of TRPV1. In fact, Buckinx and colleagues [62] found that different distribution patterns of TRPV1 in the ENS were due to the antibodies discriminating between different modulated forms of TRPV1, which influence the recognition of the intracellular forms of TRPV1. 

The large percentages of CB1R and TRPV1immunoreactive neurons, which we found in the porcine MP ileum, allowed us to speculate that CB1R and TRPV1 may co-exist on the same subclass of cholinergic neurons as substantiated by functional and immunohistochemical studies [35,63]. Double labeling of CB1R with TRPV1 (and the other receptors) was not examined due to incompatibility of the species in which the antisera were raised.

The expression of TRPV1 by enteric neurons could constitute a target for the development of new therapies against nociceptive and inflammatory intestinal stimuli [48]. Moreover, TRPV1 seems to be involved in protection against pathogenic bacteria such as *Salmonella enterica*, releasing the calcitonin gene-related peptide (CGRP), which regulates the number of microfold (M) cells and the levels of segmented filamentous bacteria that fight pathogen colonization [64]. 

The expression of TRPV1-IR in EGCs might be involved in their differentiation/maturation as suggested by Yamamoto et al. [65], or might be upregulated in different conditions of gut homeostasis/physiology as observed in the porcine tissues. The localization of TRPV1 on endothelial cells of the capillaries in the *tunica muscularis* suggested a modulation of vasocontraction and vasorelaxation in an endothelium-dependent manner, supporting the therapeutic potential of TRPV1 as a target for improving vascular functionality [66]. 

Transient potential ankyrin receptor 1 has been successfully found in the GIT neuronal [33,60,67] and non-neuronal cells [33,68,69] in which the receptor can detect specific food chemicals such as cinnamaldehyde, allyl isothiocyanate (AITC), allicin, and thymol [70,71]. In the present study, TRPA1 was observed in the enteric neurons as described in the MP neurons of rodents [33,67]. Moreover, functional investigations have indicated that TRPA1 may regulate gastrointestinal motility by means of the 5-HT release from enterochromaffin cells [72]. However, the localization of TRPA1 in the enteric neurons indicated that it could directly modulate intestinal contraction/motility, as suggested by Sandor et al. [73]. In addition, localization on the endothelial cells of the blood vessels suggested a role in controlling vasodilatation and vasoconstriction, as for TRPV1. The ability of botanicals to act on TRPA1 in the GIT seemed to modulate the majority of its functionality. In fact, AITC modulated the gastrointestinal contractions in mice [60,74], guinea-pigs [72], and dogs [75], and inhibited colonic transit [67] via TRPA1 activation. Moreover, AITC was also capable of inducing blood vessel dilatation due to the activation of TRPA1, as reported by Earley et al. [76,77] and Sullivan et al. [78]. It is possible to assume that the use of botanicals capable of modulating TRPA1 and/or TRPV1 could also play a role in reducing or controlling inflammatory stimuli. In fact, Blackshaw et al. [79] suggested that TRPA1-IR expressed by intramural neurons might not contribute to normal ENS functions, exerting its role only during inflammation or injury, or in response to exogenous agonism.

In the porcine ENS, 5-HT has been found in MP neurons of the pig colon [80] and perineuronal varicosities of the ileum [81]. Given the variety and the complexity of the effects that 5-HT exerts in the gut, it is not surprising that there is more than one type of enteric neuronal 5-HT receptor [82]. Many of the effects of CBD are mediated through 5-HT receptor activation in the CNS and peripheral nervous system, which regulate neuronal excitability and neurotransmitter release. Of the 5-HT receptors, CBD acts as an agonist on the 5-HT1aR, as a partial agonist on the 5-HT2aR, and as an antagonist on the 5-HT3R [5,83]. The full agonism of CBD at the 5-HT1aR is responsible for the anxiolytic/antidepressant and analgesic effects of CBD in animals [84,85,86].

In the present study, 5-HT1aR-IR was observed in the MP neurons and smooth muscle cells of the *tunica muscularis* in line with Youn et al. [87] and Delesalle et al. [88], who observed 5-HT1aR-IR in the MP neurons and *tunica muscularis* of the guinea-pig stomach and muscular smooth muscle cells of the equine jejunum, respectively. Electrophysiological studies have indicated that the 5-HT1aR is primarily involved in the presynaptic inhibition of transmitter release [89]. The location of 5-HT1aR-IR in varicosities around the MP neurons observed in the present study supported the idea that the receptor might be involved in the 5-HT mediated inhibition of cholinergic neurotransmission. The expression of 5-HT1aR-IR in approximately 50% of the MP neurons also suggested that some of the cells capable of producing the 5-HT1aR were cholinergic. The expression of the 5-HT1aR in the vascular smooth muscle cells of the pig ileum observed in the present study may support the direct action of 5-HT on the vascular smooth muscle. In pigs, direct vascular smooth muscle relaxation may be the predominant mechanism involved in the vasodilatation action of serotonin [90].

The localization of cannabinoid and cannabinoid-related receptors in the MP of pigs implied a possible role of phytocannabinoids and botanicals in the control and support of various gastrointestinal activities. For example, CBD has been found to act as an agonist on the 5-HT1aR, exerting a neuroprotective effect by modulating oxidative stress and inflammation [91]. Thymol also seemed to be capable of modulating the expression of the ECS in the porcine GIT [28], representing a therapeutic approach to several gastrointestinal diseases. Additional investigation is required to obtain a better understanding of the localization of the ECS receptors in the GIT of pigs including other tracts, receptors, and phenotypes of the enteric neurons in an attempt to overcome the limitations posed by the absence of specific antibodies for pigs.

## 5. Conclusions

In conclusion, the data in the present study highlighted the expression of cannabinoid (CB1R and CB2R) and cannabinoid-related receptors (TRPV1, TRPA1, and 5-HT1aR) not only in the MP neurons and enteric glial cells, but also on the smooth muscle cells and the blood vessels of the porcine ileum. These morphological findings could be of particular relevance for future functional, pre-clinical, and clinical studies assessing the effects of cannabinoids in pigs in order to manage the hypermotility associated with gastrointestinal inflammatory diseases and pain. In fact, this could justify the use of phytocannabinoids or natural molecules capable of modulating the ECS in the diet of pigs. By modulating the activation of cannabinoid and cannabinoid-related receptors, it seems possible to regulate gastrointestinal functionality at different levels. Of particular interest, TRPV1 can interfere with pathogen proliferation and, together with TRPA1, could play a role in reducing the inflammation that occurs during weaning. 

## Figures and Tables

**Table 1:** The primary antibodies used in the study.

Primary Antibodies	Host	Code	Dilution	Source
CB1R	Rabbit	ab23703	1:100	Abcam
CB2R	Rabbit	ab45942	1:200	Abcam
CB2R	Mouse	sc-293188	1:50	Santa Cruz
TRPV1	Rabbit	ACC-030	1:200	Alomone
TRPA1	Rabbit	ab58844	1:100	Abcam
5-HT1aR	Rabbit	ab85615	1:100	Abcam
HuC/HuD	Mouse	A21271	1:200	Life Technologies

**Table 2:** The secondary antibodies used in the study.

Secondary Antibodies	Host	Code	Dilution	Source
Anti-rabbit 488	Donkey	A-21206	1:1000	Thermo Fisher
Anti-rabbit 594	Donkey	ab150076	1:1000	Abcam
Anti-mouse 594	Donkey	A-21203	1:500	Thermo Fisher
Anti-mouse 488	Donkey	A-21202	1:500	Thermo Fisher

**Table 3:** Semiquantitative evaluation of the density of CB1R, CB2R, TRPV1, TRPA1, and 5-HT1aR immunoreactivity in different cellular elements (myenteric plexus neurons, nerve fibers, enteric glial cells, *tunica muscularis,* and blood vessels) of the pig ileum.

Receptors	MP Neurons	Nerve Fibers	EGCs	*Tunica Muscularis*	Blood Vessels
CB1R	C +/++	+	−	+	−
CB2R	−	−	−	−	−
TRPV1	++/+++	+/++	−/+++	−	E ++
TRPA1	C ++	++	−	−	E +++
5-HT1aR	C +	−	−	+	SMC +

The immunoreactive cells were graded as: −, negative; +, weakly stained, ++, moderately stained and +++, brightly stained. C: cytoplasmic; E: endothelium; EGCs: enteric glial cells; M: membrane; MP: myenteric plexus; SMCs: vascular smooth muscle cells.

## Data Availability

The data presented in this study are available on request from the corresponding author.

## Supplementary Materials

The following are available online at https://www.mdpi.com/2076-2615/11/2/263/s1, Figure S1: CB1R immunoreactivity in the submucosal plexus neurons of the human colon, Figure S2: CB2R immunoreactivity in the myenteric plexus neurons of the rat ileum, Figure S3: TRPV1 immunoreactivity in the myenteric plexus of the rat ileum.
