# Supplementary material for: Cannabinoid and Cannabinoid-Related Receptors in the Myenteric Plexus of the Porcine Ileum
Source: Animals (Basel). 2021 Jan 21;11(2):263. doi: 10.3390/ani11020263 (PMC7912003; doi:10.3390/ani11020263)
Supplement: Supplementary file 1 [file animals-11-00263-s001.zip › animals-1074780-supplementary/Toschi et al._Supplementary materials.pdf]

animals

MDPI

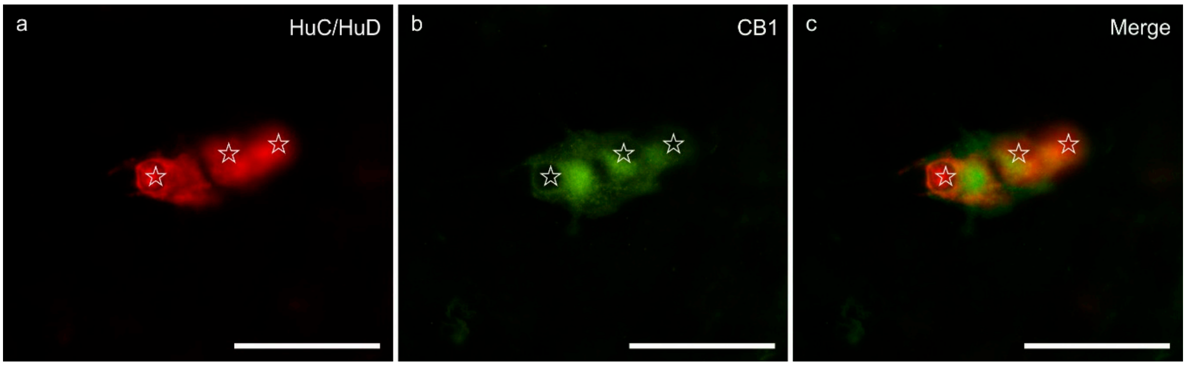

Figure S1. CB1 receptor immunoreactivity in the submucosal plexus of the human colon: (**a**) HuC/HuD immunoreactive neurons, (**b**) CB1 receptor immunoreactivity, (**c**) merge image. Stars indicate three HuC/HuD immunoreactive neurons, which co-expressed CB1 receptor immunoreactivity. Scale bar: 50 μm

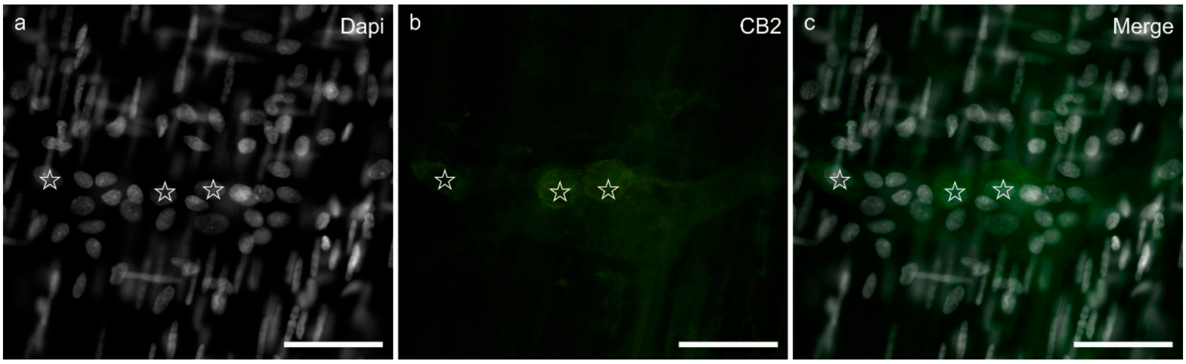

Figure S2. CB2 receptor immunoreactivity in the myenteric plexus neurons of the rat ileum: (a) Dapi stained neuronal nuclei of neurons, (**b**) CB2 receptor immunoreactivity, (**c**) merge image. Stars indicate the Dapi stained neuronal nuclei of three neurons, which co-expressed CB2 receptor immunoreactivity. Scale bar: 50 μm

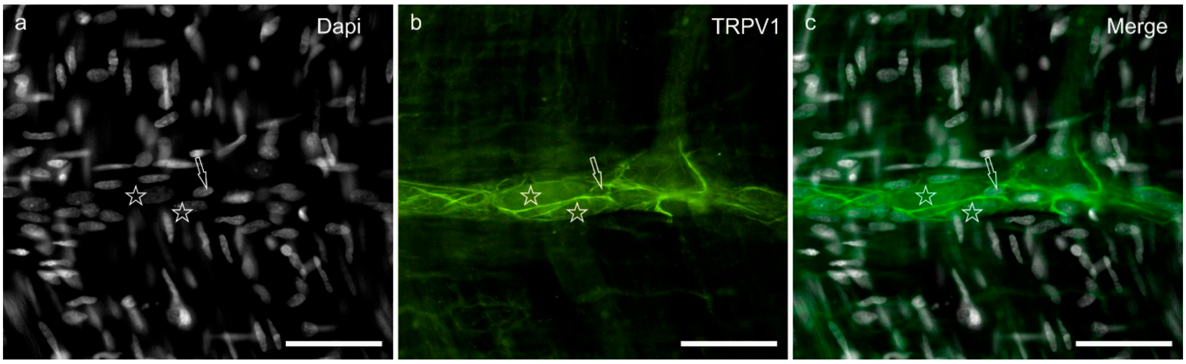

Figure S3. TRPV1 immunoreactivity in the myenteric plexus of the rat ileum: (a) Dapi stained neuronal nuclei of neurons, (**b**) TRPV1 immunoreactivity, (**c**) merge image. Stars indicate the Dapi stained neuronal nuclei of two neurons, which co-expressed weak TRPV1 immunoreactivity. The arrow indicates the nucleus of one enteric glial cell expressing bright TRPV1 immunoreactivity. Scale bar: 50 μm
