# Supplementary figures and images for: Cannabinoid and Cannabinoid-Related Receptors in the Myenteric Plexus of the Porcine Ileum
Source: Animals (Basel). 2021 Jan 21;11(2):263. doi: 10.3390/ani11020263 (PMC7912003; doi:10.3390/ani11020263)

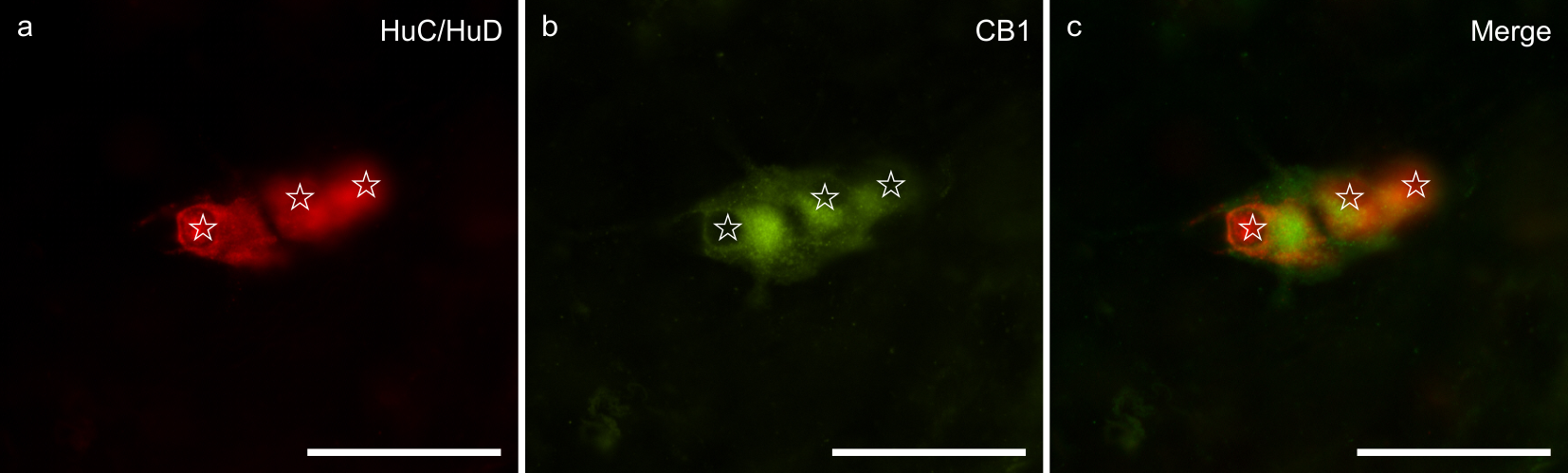

Supplement: Supplementary file 1 [file animals-11-00263-s001.zip › animals-1074780-supplementary/Figure S1.tif]

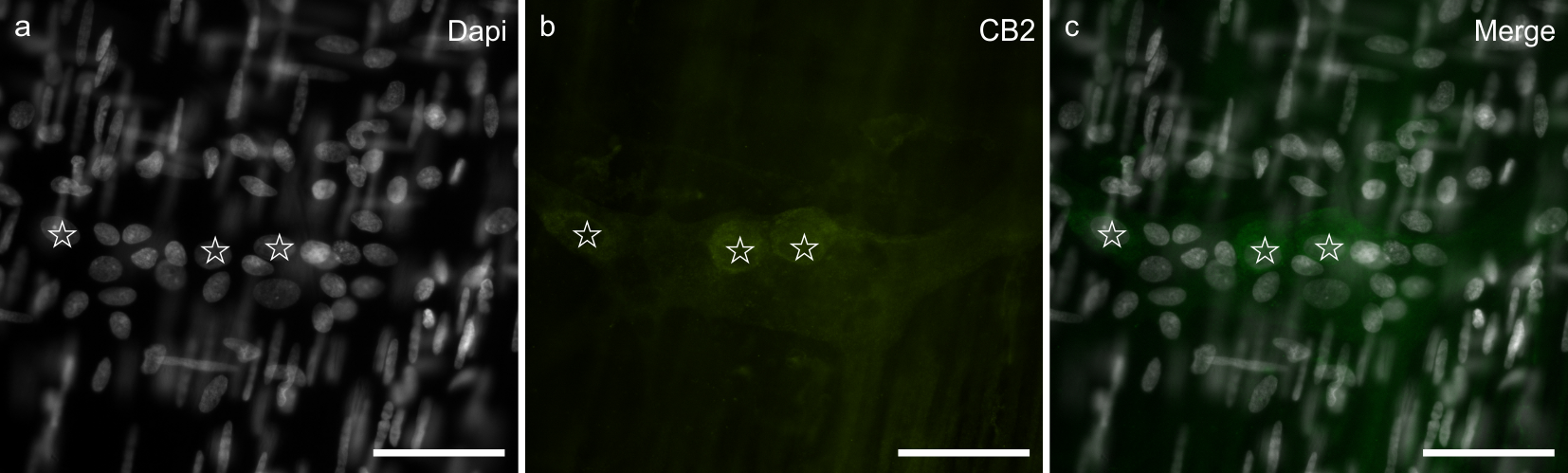

Supplement: Supplementary file 1 [file animals-11-00263-s001.zip › animals-1074780-supplementary/Figure S2.tif]

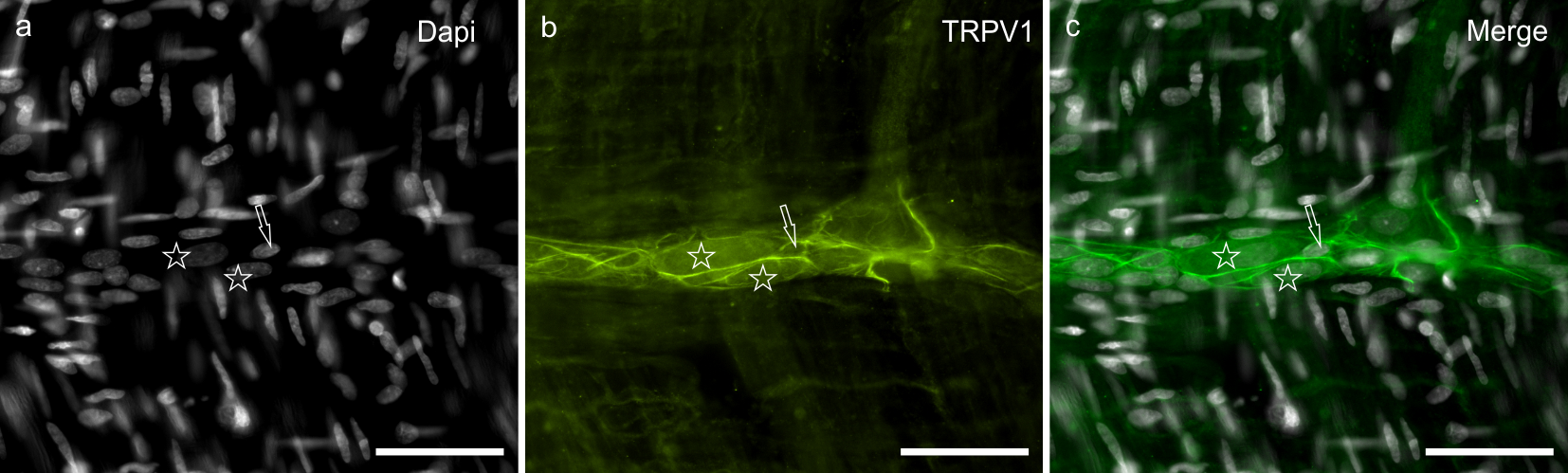

Supplement: Supplementary file 1 [file animals-11-00263-s001.zip › animals-1074780-supplementary/Figure S3.tif]
